# Comparative analyses of Linderniaceae plastomes, with implications for its phylogeny and evolution

**DOI:** 10.3389/fpls.2023.1265641

**Published:** 2023-09-26

**Authors:** Rongrong Yan, Yanfei Geng, Yuhuan Jia, Chunlei Xiang, Xinxin Zhou, Guoxiong Hu

**Affiliations:** ^1^ Key Laboratory of Plant Resource Conservation and Germplasm Innovation in Mountainous Region (Ministry of Education), Guizhou University, Guiyang, Guizhou, China; ^2^ College of Life Sciences, Guizhou University, Guiyang, Guizhou, China; ^3^ College of Tea Science, Guizhou University, Guiyang, Guizhou, China; ^4^ Key Laboratory for Plant Diversity and Biogeography of East Asia, Kunming Institute of Botany, Chinese Academy of Sciences, Kunming, Yunnan, China; ^5^ South China Botanical Garden, Chinese Academy of Sciences, Guangzhou, Guangdong, China

**Keywords:** Lindernieae, *Lindernia*, *Picria*, *Torenia*, Scrophulariaceae, chloroplast genome, phylogenomics

## Abstract

**Introduction:**

The recently established Linderniaceae, separated from the traditionally defined Scrophulariaceae, is a taxonomically complicated family. Although previous phylogenetic studies based on a few short DNA markers have made great contributions to the taxonomy of Linderniaceae, limited sampling and low resolution of the phylogenetic tree have failed to resolve controversies between some generic circumscriptions. The plastid genome exhibits a powerful ability to solve phylogenetic relationships ranging from shallow to deep taxonomic levels. To date, no plastid phylogenomic studies have been carried out in Linderniaceae.

**Methods:**

In this study, we newly sequenced 26 plastid genomes of Linderniaceae, including eight genera and 25 species, to explore the phylogenetic relationships and genome evolution of the family through plastid phylogenomic and comparative genomic analyses.

**Results:**

The plastid genome size of Linderniaceae ranged from 152,386 bp to 154,402 bp, exhibiting a typical quartile structure. All plastomes encoded 114 unique genes, comprising 80 protein-coding genes, 30 tRNA genes, and four rRNA genes. The inverted repeat regions were more conserved compared with the single-copy regions. A total of 1803 microsatellites and 1909 long sequence repeats were identified, and five hypervariable regions (*petN-psbM*, *rps16-trnQ*, *rpl32-trnL*, *rpl32*, and *ycf1*) were screened out. Most protein-coding genes were relatively conserved, with only the *ycf2* gene found under positive selection in a few species. Phylogenomic analyses confirmed that Linderniaceae was a distinctive lineage and revealed that the presently circumscribed *Vandellia* and *Torenia* were non-monophyletic.

**Discussion:**

Comparative analyses showed the Linderniaceae plastomes were highly conservative in terms of structure, gene order, and gene content. Combining morphological and molecular evidence, we supported the newly established *Yamazakia* separating from *Vandellia* and the monotypic *Picria* as a separate genus. These findings provide further evidence to recognize the phylogenetic relationships among Linderniaceae and new insights into the evolution of the plastid genomes.

## 1 Introduction

As traditionally circumscribed, *Lindernia* and its relatives were placed in the tribe Lindernieae (Reichenbach, 1831) of Scrophulariaceae. The family is characterized by bilaterally symmetric and often tubular flowers, ovaries with axile placentation, numerous ovules, and capsules, which are shared with other related families of Lamiales (Bentham, 1846; Wettstein, 1891; Olmstead et al., 2001; Fischer, 2004). The absence of morphological synapomorphy raises the suspicion that the traditionally defined Scrophulariaceae is not monophyletic, with evidence from molecular phylogenetic analyses (Olmstead and Reeves, 1995; Olmstead et al., 2001; Beardsley and Olmstead, 2002; Oxelman et al., 2005). Combining molecular with morphological evidence, the tribe Lindernieae was promoted to the family Linderniaceae by Rahmanzadeh et al. (2005). Thereafter, Linderniaceae as an independent lineage distinct from Scrophulariaceae has been widely accepted in the comprehensive reviews of Lamiales (Tank et al., 2006; Angiosperm Phylogeny Group (APG), 2009; Schäferhoff et al., 2010; Refulio-Rodriguez and Olmstead, 2014; Angiosperm Phylogeny Group (APG), 2016; Liu et al., 2020; Fonseca, 2021). The family is characterized by special abaxial stamens with filaments conspicuously geniculate, zig-zag-shaped, or bearing spur-like appendages. The diversity centers of Linderniaceae are situated in tropical Africa and Southeast Asia, with 22 genera and more than 220 species recorded (Rahmanzadeh et al., 2005; Tank et al., 2006; Fischer et al., 2013; Perret et al., 2013; Biffin et al., 2018; Almeida et al., 2019; Yan et al., 2023).

Historically, diagnostic characteristics of genera of the Linderniaceae have been controversial among different taxonomists, which results in ambiguous generic boundaries. For example, Bentham (1846) considered *Lindernia* and the other three closely related genera (*Bonnaya*, *Ilysanthes*, and *Vandellia*) as four distinct genera. Based on the number of fertile stamens, Urban (1884) and Wettstein (1891) included *Bonnaya* with two fertile stamens into *Ilysanthes* and *Vandellia* with four fertile stamens into *Lindernia*. However, Haines (1922) emphasized the diagnostic value of the leaf vein, reducing *Bonnaya* with pinnate vein to *Vandellia* and *Ilysanthes* with palmate vein to *Lindernia*. Pennell (1935) suggested that the diagnostic characteristics currently used were too weak and artificial, and included the four controversial genera into a broadly circumscribed genus *Lindernia* sensu lato (s.l.). Synapomorphies of the *Lindernia* sensu Pennell (1935) comprise the remarkably uniform corolla, curved anterior filaments, and similar septicidal dehiscence of the capsule. The treatment was followed by the majority of botanists (Philcox, 1968; Yamazaki, 1977; Yamazaki, 1985; Fischer, 1995; Lewis, 2000; Fischer, 2004; Rahmanzadeh et al., 2005). However, the *Lindernia* s.l. seems not to be a natural group as some species incorporated into the *Lindernia* s.l. have the same characteristics as calyx, staminal appendages, and floral disc of another genus *Torenia* (Hara, 1943; Pennell, 1943; Philcox, 1968). On the basis of the morphology of leaf margin and seed, Yamazaki (1954; 1955) divided *Lindernia* s.l. into *Lindernia* s.s. (entire or slightly dentate leaf margin and non-alveolated seed) and *Vandellia* (serrate leaf margin and bothrospermous seed), although the revision has not been widely accepted. On the other hand, there were complicated relationships between *Torenia* and its relatives. The genus *Craterostigma* mainly distributed in Africa was reduced to a section of *Torenia* by bearing the winged calyx (Bentham, 1846). Wettstein (1891) supported *Craterostigma* as a separate genus based on its rosulate habit, but *Craterostigma* species often were described as *Torenia* in subsequent investigations (Schlechter, 1924). Since traditional classifications failed to provide a stable taxonomic framework at the generic level, evidence from the molecular level has been given weight.

When establishing Linderniaceae, Rahmanzadeh et al. (2005) included 13 genera of the tribe Lindernieae sensu Fischer (2004) within this family, of which the type genus *Lindernia* is the largest genus comprising ca. 100 species. Using two DNA markers (*trnK* and *matK*), Fischer et al. (2013) revealed the polyphyly of *Lindernia* and elaborated the first generic classification for Linderniaceae. In this study, 17 genera were recognized, of which *Bonnaya* and *Vandellia* were reinstated from *Lindernia* s.l., and formerly well-circumscribed *Torenia* and *Craterostigma* were expanded to include some members of *Lindernia* s.l. (Fischer et al., 2013). The narrowly circumscribed *Lindernia* was kept according to the delimitation of Yamazaki (1955), including *Bryodes*, *Ilysanthes*, and *Psammetes*. In Fischer et al. (2013) treatment, the species not represented by molecular data were assigned to genera according to morphology, of which most taxa with pinnate veins and deeply lobed calyx from Asia and America were transferred to *Vandellia*. On the basis of expanded phylogenetic sampling and four DNA markers (*rps16*, *matK*, *trnL*-*F*, and *RPB2*), however, Liang (2017) revealed the polyphyly of *Vandellia* sensu Fischer et al. (2013) as other long-accepted genera (e.g., *Torenia*, *Craterostigma*, *Chamaegigas*, and *Linderniella*) embedded. Based on phylogenetic analyses inferred from the *matK* marker, Biffin et al. (2018) narrowed *Vandellia* sensu Fischer et al. (2013) and formally established the new genus *Yamazakia* separating from *Vandellia*. Although molecular phylogenetic studies have made great contributions to the circumscription of Linderniaceae, there are still considerable controversies concerning generic circumscription, predominantly due to the limited sampling and the low resolution of phylogenetic trees inferred from a few short molecular markers.

With the rapid development of Next-generation sequencing (NGS) technology, more and more organelle genomes were sequenced and submitted to GenBank. Compared to nuclear and mitochondrial genomes, the plastid genome has been widely applied to resolve phylogenetic relationships at different taxonomic levels, owing to its relatively small genome and slow mutation rate (Parks et al., 2009; Wicke et al., 2011; Wei et al., 2017; Wen et al., 2021; Zhao et al., 2021; Dong et al., 2022). The complete plastome exhibits a powerful ability to solve backbone phylogeny in contrast with universal DNA markers (Alwadani et al., 2019; Chen et al., 2022; Peng et al., 2023). Moreover, comparative analyses of the plastid genomes not only provide plentiful information for phylogenetic relationships but also deepen our understanding of genome evolution (Chu et al., 2022; Ogoma et al., 2022; Wan et al., 2023). However, no comparative analyses related to Linderniaceae plastomes have been conducted so far, only general reports about genome size and gene contents (Cheng et al., 2019; Chen et al., 2021). In this study, for the first time, we attempted to explore the phylogenetic relationships and genome evolution of Linderniaceae using a large number of complete plastomes. Our goals were to (1) investigate the structure and variation of Linderniaceae plastomes, (2) provide insights into the evolution of Linderniaceae plastomes, and (3) infer the phylogeny of Linderniaceae based on plastid genomic data.

## 2 Materials and methods

### 2.1 Plant material, DNA extraction, and sequencing

In total, we included 31 samples of Linderniaceae representing eight genera and 28 species, of which 26 individuals were newly sequenced and the other five species were downloaded from GenBank (**Table 1**). Scientific names of Linderniaceae followed by Fischer et al. (2013) and Biffin et al. (2018). Plant samples were collected from the field and fresh leaves from healthy plants were stored in silica gel. These voucher specimens were deposited in the Herbarium of the Natural Museum of Guizhou University (GACP) (**Table 1**). The modified CTAB method was employed to extract total genomic DNA (Doyle and Doyle, 1987). The DNA purity and concentration were quantified using agarose gel electrophoresis and a NanoDrop 2000 Spectrophotometer. The quantified DNA was used to construct shotgun libraries with fragments of 200–500 bp, and paired-end (150 bp) reads were sequenced using an Illumina Hiseq 2500 platform by Biomarker Technologies, Inc (Shandong, China). Low-quality sequences of raw reads were filtered using the software Trimmomatic v.0.32 (Bolger et al., 2014), yielding at least 3 GB of clean reads for each sample.

**Table 1:** Voucher information and GenBank accession numbers of the samples in this study.

Taxa	Locality	Vouchers	GenBank accession
*Bonnaya antipoda* (L.) Druce	Guiping, Guangxi, China	Yan et al. YRR003	**OQ784229**
*Bonnaya ciliata* (Colsm.) Spreng.	Guigang, Guangxi, China	Yan et al. YRR010	**OQ784230**
*Bonnaya ruellioides* (Colsm.) Spreng.	Jiujiang, Jiangxi, China	Hu et al. GX Hu 700	**OQ784231**
*Bonnaya tenuifolia* (Colsm.) Spreng.	Hangzhou, Zhejiang, China	Hu et al. GX Hu 719	**OQ784232**
*Craterostigma nummulariifolium* (D. Don) Eb. Fisch., Schäferh. & Kai Müll.	Hangzhou, Zhejiang, China	Hu et al. GX Hu 724	**OQ808813**
*Legazpia polygonoides* (Benth.) Yamazaki 1	Guangzhou, Guangdong, China	Zhou et al. LSX765	**OQ808814**
*Legazpia polygonoides* (Benth.) Yamazaki 2	Guigang, Guangxi, China	Yan et al. YRR013	**OQ808815**
*Legazpia polygonoides* (Benth.) Yamazaki	NA	NA	OP066243
*Lindernia procumbens* (Krock.) Borbas	Hangzhou, Zhejiang, China	Hu et al. GX Hu 720	**OQ808816**
*Lindernia rotundifolia* (L.) Alston	Xishuangbanna, Yunnan, China	Cao et al., 2022017	**OQ808817**
*Picria felterrae* Lour.	Wuzhishan, Hainan, China	Zhou et al. LSX796	**OQ808818**
*Picria felterrae* Lour.	NA	NA	NC_065864
*Torenia anagallis* (Burm.f.) Wannan, W.R.Barker & Y.S.Liang	Jiujiang, Jiangxi, China	Hu et al. GX Hu 698	**OQ808819**
*Torenia asiatica* L.	Laibin, Guangxi, China	Yan et al. YRR024	**OQ808820**
*Torenia benthamiana* Hance	Gaozhou, Guangdong, China	LNH180702030	NC_045273
*Torenia concolor* Lindl.	Libo, Guizhou, China	Hu et al. GX Hu 727	**OQ808821**
*Torenia crustacea* (L.) Cham. & Schltdl.	Jiujiang, Jiangxi, China	Hu et al. GX Hu 697	**OQ808822**
*Torenia flava* Buch.-Ham. ex Benth.	Guiping, Guangxi, China	Yan et al. YRR020	**OQ808823**
*Torenia fordii* Hook. f.	NA	NA	MW309811
*Torenia fournieri* Linden. ex Fourn.	Zhaoqing, Guangdong, China	BGCLSZU001	NC_056129
*Torenia oblonga* (Benth.) Hance	Guiping, Guangxi, China	Yan et al. YRR008	**OQ808824**
*Torenia parviflora* Ham. ex Benth.	Laibin, Guangxi, China	Yan et al. YRR022	**OQ808825**
*Torenia violacea* (Blanco) Pennell	Laibin, Guangxi, China	Yan et al. YRR023	**OQ808826**
*Vandellia elata* Benth.	Guigang, Guangxi, China	Yan et al. YRR012	**OQ808827**
*Vandellia megaphylla* (P. C. Tsoong) Eb. Fisch., Schäferh. & Kai Müll.	Wangmo, Guizhou, China	Hu et al. GX Hu 749	**OQ808828**
*Vandellia montana* (Blume) Benth.	Lingshui, Hainan, China	Zhou et al., 2022009	**OQ808829**
*Vandellia scutellariiformis* (T. Yamaz.) T. Yamaz.	Xinyi, Guangdong, China	Zhou et al., 2022016	**OQ808830**
*Vandellia setulosa* (Maxim.) T. Yamaz.	Nanping, Fujiang, China	Hu et al. GX Hu 711	**OQ808832**
*Vandellia stricta* (P. C. Tsoong & T. C. Ku) Eb. Fisch., Schäferh. & Kai Müll.	Guiping, Guangxi, China	Yan et al. YRR018	**OQ808831**
*Yamazakia pusilla* (Willd.) W.R.Barker, Y.S.Liang & Wannan	Laibin, Guangxi, China	Yan et al. YRR006	**OQ808833**
*Yamazakia viscosa* (Hornem.)W.R. Barker, Y.S.Liang & Wannan	Laibin, Guangxi, China	Yan et al. YRR001	**OQ808834**
*Scrophularia cephalantha* Nakai.	Tongyeong-si, Gyeongsangnam-do, Korea	HSN12443	MN255822
*Scrophularia ningpoensis* Hemsl.	Jianshi, Hubei, China	H. D. Jang 504	MN734369
*Plantago media* L.	NA	NA	NC_028520

GenBank accession numbers of the newly sequenced were shown in bold, other sequences were from the previous studies (NA, information is unavailable).

### 2.2 Genome assembly, validation, and annotation

*De novo* assemblies of the plastomes were conducted with GetOrganelle v1.7.5, following default settings (Jin et al., 2020). The final assembly results were verified using Bandage v0.8.1 (Wick et al., 2015). Two annotation tools, PGA (Qu et al., 2019) and CPGAVAS2 (Shi et al., 2019), were used to annotate these plastomes with *Torenia benthamiana* (NC_045273) as a reference. After the initial annotation, tRNA genes were further checked by tRNAscan-SE v1.21 (Brooks and Lowe, 2005). We manually calibrated the start and stop codons of coding sequences in the software Geneious v9.0.2 (Kearse et al., 2012). The gene maps were drawn using OrganellarGenomeDRAW (OGDRAW) (Lohse et al., 2013). All the newly sequenced plastomes were deposited at GenBank with accession numbers in **Table 1**.

### 2.3 Plastome comparative analyses

Gene rearrangements were detected based on collinear blocks using Mauve v2.4.0 (Darling et al., 2004). Genome divergence was identified using mVISTA with Shuffle-LAGAN mode (Frazer et al., 2004). To detect hypervariable regions, nucleotide variability (Pi) was calculated in DnaSP v.5.0 (Librado and Rozas, 2009) with the parameters of 600 bp on window length and 200 bp on step size. We also compared the boundaries of large single copy (LSC), small single copy (SSC), and two inverted repeat (IR) regions by online program IRscope (Amiryousefi et al., 2018).

### 2.4 Repeat sequences analyses

Simple sequence repeats (SSRs) were identified in MISA-web (Beier et al., 2017), with parameters set to ten, five, four, three, three, and three for mono-, di-, tri-, tetra-, penta-, and hexa-nucleotides, respectively. The forward, reverse, palindromic, and complement repeats were recognized using REPuter (Kurtz et al., 2001), with a minimum repeat size of 30 bp and a Hamming distance of three. The repeat numbers in the regions of LSC, SSC, and IR were counted in Geneious v9.0.2 (Kearse et al., 2012).

### 2.5 Condon usage and selective pressure analyses

The codon usage bias of protein-coding genes based on 28 Linderniaceae species was calculated in CodonW v1.4.4 (Peden, 2000). Relative synonymous codon usage (RSCU) was used to evaluate codon usage preference. When the RSCU value > 1 indicates that the codon is preferred, the RSCU value = 1 means that the codon is not preferred, and the RSCU value < 1 shows that the codon usage is low. The effective number of codons (ENC) and GC content of the synonymous third codon positions (GC3s) were used to assess codon usage patterns. To investigate the selective pressure on protein-coding genes, we extracted the shared nucleotide and protein sequences based on 28 Linderniaceae species using Geneious v9.0.2 (Kearse et al., 2012). Protein sequences were aligned by MAFFT v.7.388 (Katoh and Standley, 2013), and then transformed data into axt format. Non-synonymous (Ka) substitution and synonymous (Ks) substitution were calculated using KaKs_Calculator v.2.0 (Wang et al., 2010), with *Lindernia procumbens* as the reference, setting the genetic code to 11 (bacterial and plant plastid codes) and calculation model to Yang-Nielsen algorithm (Nielsen and Yang, 1998). The ratio of non-synonymous and synonymous substitutions (Ka/Ks) is greater than 1, equal to 1, and less than 1, indicating positive selection, neutral selection, and purifying selection, respectively. The Ka value of 0 showed that the Ka/Ks value equaled 0. Ka = 0 and Ks = 0 showed the invalid value was represented by NA.

### 2.6 Phylogenetic analyses

Phylogenetic relationships of Linderniaceae were reconstructed using plastid genomic data (complete plastid genomes and 80 protein-coding genes). The ingroup included 31 individuals of Linderniaceae, representing eight genera and 28 species, with three species from Plantaginaceae and Scrophulariaceae as the outgroups (**Table 1**). Alignments of sequences were performed in MAFFT v.7.388 with auto strategy (Katoh and Standley, 2013) and manually adjusted the alignment results in PhyDE v.0.9971 (Müller et al., 2010). Phylogenetic trees were conducted using maximum likelihood (ML) and Bayesian inference (BI) methods in CIPRES Science Gateway (https://www.phylo.org/) with the tool of RAxML-HPC2 on XSEDE 8.2.12 and MrBayes on XSEDE 3.2.7a, respectively. The ML tree was inferred under the GTRGAMMA model, with bootstrap replicates of 1,000. For BI analysis, the best-fit model was selected in the software PhyloSuite (Zhang et al., 2020). The GTR+F+I+G4 model was selected for two data sets under the Akaike Information Criterion (AIC) (David and Buckley, 2004). Two Markov Chain Monte Carlo (MCMC) simulations were run for 2,000,000 generations independently, with every 1,000 generations for tree sampling. The initial 25% of trees were removed as burn-in and the remaining data were used to generate consensus trees. Convergences were inspected when the average standard deviation of the split frequencies was less than 0.001. Finally, we visualized and edited these trees using iTOL v3.4.3 (Letunic and Bork, 2016).

## 3 Results

### 3.1 Structure and features of Linderniaceae plastomes

**Figure 1:**
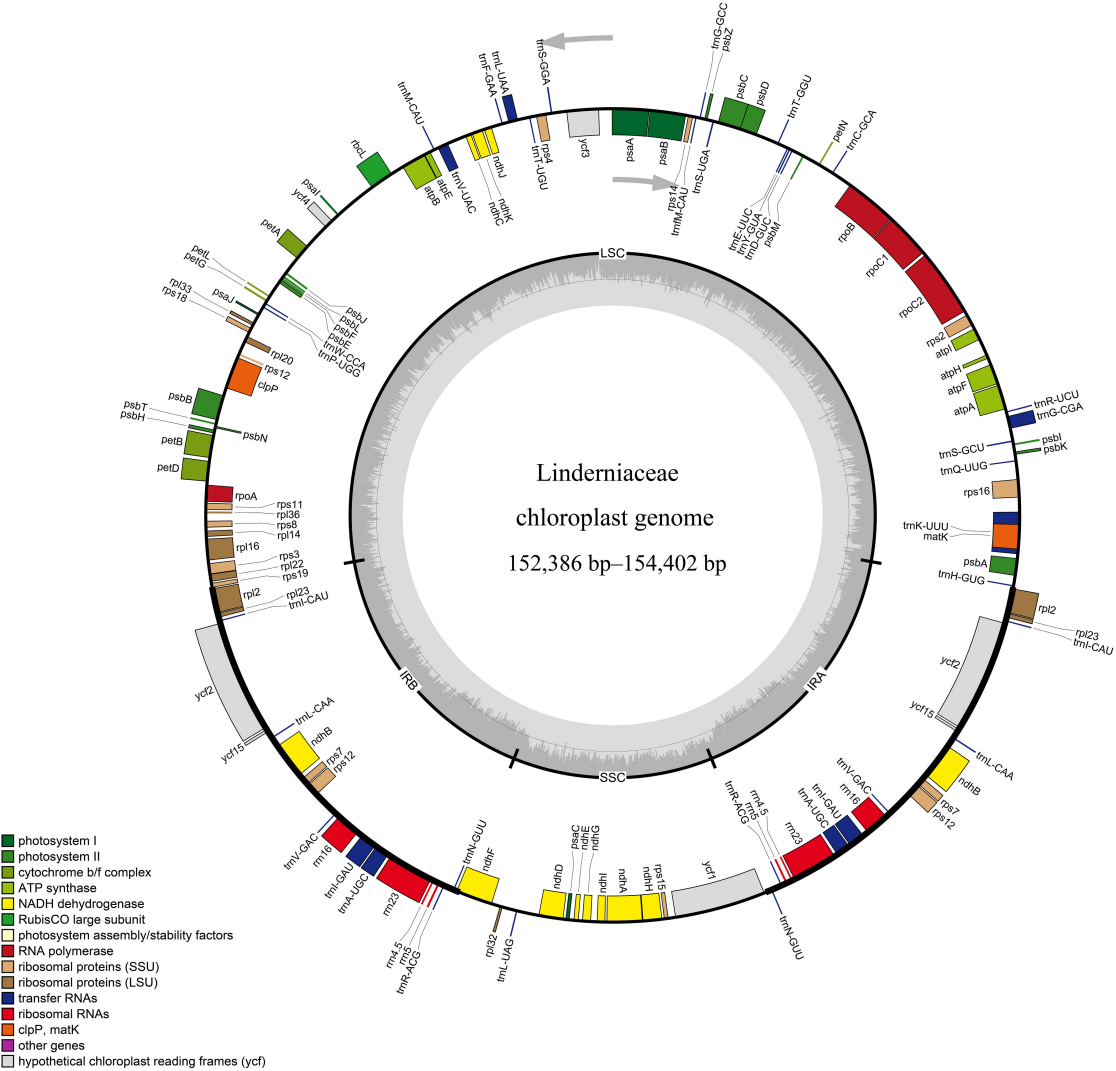
Representative circular map of the Linderniaceae plastome. Genes inside the circle are transcribed clockwise, while those outside the circle are transcribed counter-clockwise. The genes are color-coded with different functions. The darker gray and lighter gray in the inner correspond to GC content and AT content, respectively. LSC, large single copy; IR, inverted repeat; SSC, small single copy.

The whole plastid genomes of 31 Linderniaceae ranged from 152,386 bp (*Bonnaya ciliata*) to 154,402 bp (*Yamazakia viscosa*) (**Table 2**). These plastomes displayed a typical quadripartite structure (**Figure 1**), with a large single copy (LSC) region, a small single copy (SSC) region, and a pair of inverted repeats (IRs). The size of the LSC region varied from 84,566 bp in *B. ruellioides* to 85,837 bp in *Y. viscosa*. The size of the SSC region was from 18,352 bp in *B. ciliata* to 19,095 bp in *Lindernia procumbens*. The size of the IR region ranged from 24,599 bp in *L. procumbens* to 24,873 bp in *Vandellia scutellariiformis*. Total GC content did not present significant differences among all plastomes (37.5–37.7%), with 35.3–35.6% in the LSC region, 31.6–32.0% in the SSC region, and 43.3–43.6% in the IR region. Gene content was identical in all Linderniaceae plastomes, with 114 unique genes, comprising 80 protein-coding genes (PCGs), 30 tRNA genes, and four rRNA genes (**Table 3**). Seven protein-coding genes, seven tRNA genes, and four rRNA genes were duplicated in the IR regions. A total of 17 genes with introns were detected, of which *clpP*, *rps12*, and *ycf3* genes contained two introns and the other 14 genes had a single intron. With the first exon situated in the LSC region and the remaining two exons dispersed throughout the IR regions, the *rps12* was identified as a trans-spliced gene.

**Table 2:** Summary of features of the Linderniaceae plastomes.

Taxa	Length (bp)				GC content (%)				Unique genes				Total genes
	Total	LSC	SSC	IR	Total	LSC	SSC	IR	Total	PCGs	tRNA	rRNA	
*Bonnaya antipoda*	152,815	84,598	18,751	24,733	37.5	35.4	31.8	43.4	114	80	30	4	132
*Bonnaya ciliata*	152,386	84,606	18,352	24,714	37.5	35.4	31.8	43.3	114	80	30	4	132
*Bonnaya ruellioides*	152,706	84,566	18,772	24,684	37.5	35.4	31.6	43.4	114	80	30	4	132
*Bonnaya tenuifolia*	152,767	84,632	18,723	24,706	37.5	35.4	31.7	43.4	114	80	30	4	132
*Craterostigma nummulariifolium*	153,028	85,234	18,352	24,721	37.5	35.3	31.7	43.3	114	80	30	4	132
*Legazpia polygonoides*1	153,477	85,055	18,858	24,782	37.7	35.6	31.9	43.5	114	80	30	4	132
*Legazpia polygonoides*2	153,477	85,055	18,858	24,782	37.7	35.6	31.9	43.5	114	80	30	4	132
Legazpia polygonoides*	153,477	85,055	18,864	24,779	37.7	35.6	31.9	43.5	114	80	30	4	132
*Lindernia procumbens*	153,448	85,155	19,095	24,599	37.7	35.6	31.9	43.6	114	80	30	4	132
*Lindernia rotundifolia*	153,360	85,094	19,014	24,626	37.6	35.6	31.8	43.5	114	80	30	4	132
*Picria felterrae*	153,528	85,400	18,632	24,748	37.5	35.4	31.7	43.4	114	80	30	4	132
*Picria felterrae* *	153,521	85,390	18,635	24,748	37.5	35.4	32.0	43.5	114	80	30	4	132
*Torenia anagallis*	153,459	85,142	18,803	24,757	37.6	35.4	32.0	43.4	114	80	30	4	132
*Torenia asiatica*	154,006	85,653	18,803	24,775	37.5	35.3	32.0	43.5	114	80	30	4	132
Torenia benthamiana*	153,526	85,417	18,833	24,638	37.6	35.4	32.0	43.6	114	80	30	4	132
*Torenia concolor*	153,994	85,642	18,802	24,775	37.5	35.3	32.0	43.5	114	80	30	4	132
*Torenia crustacea*	153,668	85,432	18,724	24,756	37.6	35.4	32.0	43.5	114	80	30	4	132
*Torenia flava*	153,873	85,565	18,814	24,747	37.6	35.4	32.0	43.5	114	80	30	4	132
Torenia fordii*	154,007	85,559	18,830	24,809	37.6	35.4	32.0	43.5	114	80	30	4	132
Torenia fournieri*	153,938	85,498	18,830	24,805	37.6	35.4	32.0	43.5	114	80	30	4	132
*Torenia oblonga*	153,669	85,433	18,724	24,756	37.6	35.4	32.0	43.5	114	80	30	4	132
*Torenia parviflora*	153,013	84,800	18,763	24,725	37.6	35.4	32.0	43.5	114	80	30	4	132
*Torenia violacea*	153,970	85,558	18,830	24,791	37.6	35.4	32.0	43.5	114	80	30	4	132
*Vandellia elata*	152,900	84,806	18,452	24,821	37.7	35.5	32.0	43.4	114	80	30	4	132
*Vandellia megaphylla*	153,991	85,571	18,750	24,835	37.6	35.5	32.0	43.4	114	80	30	4	132
*Vandellia montana*	154,296	85,757	18,813	24,863	37.5	35.4	31.8	43.4	114	80	30	4	132
*Vandellia scutellariiformis*	153,942	85,408	18,788	24,873	37.5	35.4	31.8	43.4	114	80	30	4	132
*Vandellia setulosa*	153,688	85,226	18,762	24,850	37.6	35.4	31.8	43.4	114	80	30	4	132
*Vandellia stricta*	153,885	85,581	18,806	24,749	37.6	35.4	32.0	43.5	114	80	30	4	132
*Yamazakia pusilla*	153,876	85,399	18,771	24,853	37.5	35.4	31.7	43.4	114	80	30	4	132
*Yamazakia viscosa*	154,402	85,837	18,881	24,842	37.6	35.5	31.9	43.4	114	80	30	4	132

*, plastome was downloaded from GenBank.

**Table 3:** Gene content of the Linderniaceae plastomes.

Category	Group of genes	Genes name
Photosynthesis	Subunits of photosystem I	*psaA, psaB, psaC, psaI, psaJ*
	Subunits of photosystem II	*psbA, psbB, psbC, psbD, psbE, psbF, psbH, psbI, psbJ, psbK, psbL, psbM, psbN, psbT, psbZ*
	Subunits of NADH dehydrogenase	*ndhA* ^a^ *, ndhB* ^a^ (2)*, ndhC, ndhD, ndhE, ndhF, ndhG, ndhH, ndhI, ndhJ, ndhK*
	Subunits of cytochrome b/f complex	*petA, petB* ^a^ *, petD* ^a^ *, petG, petL, petN*
	Subunits of ATP synthase	*atpA, atpB, atpE, atpF* ^a^ *, atpH, atpI*
	Large subunit of rubisco	*rbcL*
Self-replication	Proteins of large ribosomal subunit	*rpl2* ^a^ (2)*, rpl14, rpl16* ^a^ *, rpl20, rpl22, rpl23* (2)*, rpl32, rpl33, rpl36*
	Proteins of small ribosomal subunit	*rps2, rps3, rps4, rps7* (2)*, rps8, rps11, rps12* ^b,c^ (2)*, rps14, rps15, rps16^a^, rps18, rps19*
	Subunits of RNA polymerase	*rpoA, rpoB, rpoC1^a^, rpoC2*
	Ribosomal RNAs	*rrn16* (2)*, rrn23* (2)*, rrn4.5* (2)*, rrn5* (2)
	Transfer RNAs	*trnA-UGC* ^a^ (2*), trnC-GCA, trnD-GUC, trnE-UUC, trnF-GAA, trnG-CGA* ^a^ *, trnG-GCC, trnH-GUG, trnI-CAU(2), trnI-GAU* ^a^ (2)*, trnK-UUU* ^a^ *, trnL-CAA* (2)*, trnL-UAA* ^a^ *, trnL-UAG, trnM-CAU, trnN-GUU* (2)*, trnP-UGG, trnQ-UUG, trnR-ACG* (2)*, trnR-UCU, trnS-GCU, trnS-GGA, trnS-UGA, trnT-GGU, trnT-UGU, trnV-GAC* (2)*, trnV-UAC* ^a^ *, trnW-CCA, trnY-GUA, trnfM-CAU*
Other genes	Maturase	*matK*
	Protease	*clpP* ^b^
	Envelope membrane protein	*cemA*
	Acetyl-CoA carboxylase	*accD*
	c-type cytochrome synthesis gene	*ccsA*
	Translation initiation factor	*infA*
Unknown function	Conserved hypothetical chloroplast ORF	*ycf1, ycf2* (2)*, ycf3* ^b^ *, ycf4, ycf15* (2)

^a,^ Gene with one intron.

^b,^ Gene with two introns.

^c,^ Trans-spliced gene.

(2), Gene with two copies.

Gene order in Linderniaceae was highly conservative and we did not find rearrangement or inversion events (**Figure 1** and **Supplementary Figure 1**). However, the junctions between single copy (SC) and IR regions exhibited slight differences (**Supplementary Figure 2**). The boundary of LSC and IRb was located in the intergenic region between *rps19* and *rpl2* among *Bonnaya* and *Torenia anagallis* plastomes, and the *rps19* gene crossed the LSC-IRb boundary for the other plastomes. Except for *Lindernia procumbens* and *L. rotundifolia*, the gene *ndhF* spanned the SSC-IRb junction in all taxa, with 36 bp to 240 bp in the IRb region and 2019 bp to 2165 bp in the SSC region. At the SSC-IRa junction, the *ycf1* and *trnN* genes were entirely located within the SSC and IRa region near the junction, respectively. For all species, the boundary of IRa and LSC was placed in the intergenic region between *rpl2* and *trnH*, with gene *rpl2* being 41 to 110 bp and gene *trnH* being 3 to 44 bp away from the boundary.

### 3.2 Sequence variation analyses

The mVISTA analyses indicated that the plastid genomes of Linderniaceae were relatively conservative (**Supplementary Figure 3**). The alignment of the whole plastomes was 143,491 bp in length, comprising 14,984 variable sites (10.44%) and 7,949 parsimony-informative sites (5.54%) (**Table 4**). The highest percentage of parsimony-informative sites was from the SSC region (1,739, 9.84%), and the lowest was from the IR region (300, 1.26%). There was nucleotide variability (Pi) of 0.01737 across the complete plastome. The IR region (Pi = 0.0042) displayed the lowest sequence divergence, while the SSC region (Pi = 0.03225) had the most variance.

**Table 4:** Sequence divergence of the Linderniaceae plastomes.

Regions	Aligned length (bp)	Variable sites		Information sites		Nucleotide Diversity
		Numbers	%	Numbers	%	
LSC	77,808	10,353	13.31	5,394	6.93	0.02256
SSC	17,664	3,158	17.88	1,739	9.84	0.03225
IR	23,901	710	2.97	300	1.26	0.0042
Whole plastome	143,491	14,984	10.44	7,949	5.54	0.01737

**Figure 2:**
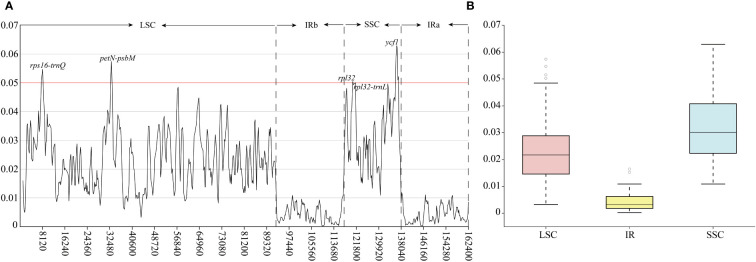
Nucleotide diversity (Pi) values of the Linderniaceae plastomes. **(A)** The Pi-value of the windows. **(B)** Boxplots of Pi-value differences among the LSC, IR, and SSC regions.

**Figure 3:**
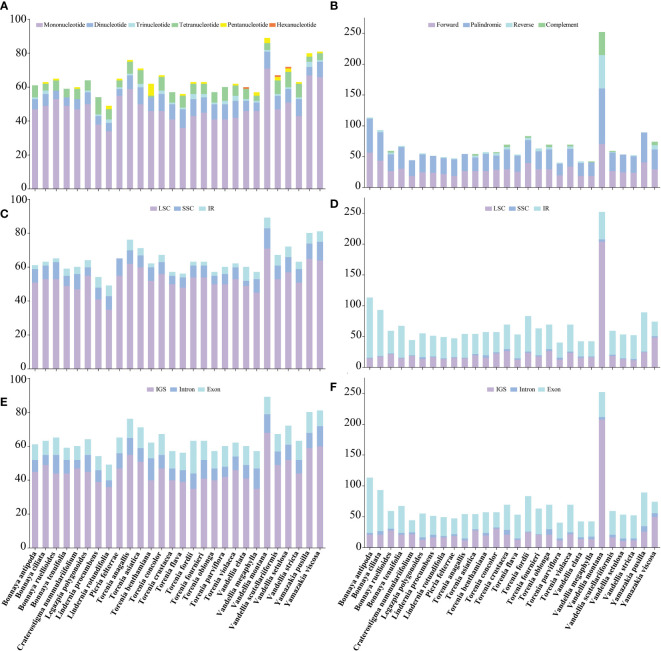
Comparisons of simple sequence repeats (SSRs) and long sequence repeats (LSRs) among 28 Linderniaceae species. **(A)** Number of six types of SSRs. **(B)** Number of four types of LSRs. **(C)** Number of SSRs in the LSC, SSC, and IR regions. **(D)** Number of LSRs in the LSC, SSC, and IR regions. **(E)** Number of SSRs in the IGS, exons, and introns. **(F)** Number of LSRs in the IGS, exons, and introns.

Hypervariable regions of Linderniaceae species were further identified through the slide window analyses (**Figure 2**). The nucleotide diversity values in plastid genomes ranged from 0.00024 to 0.06292. The mutational hotspots were defined with Pi values greater than 0.0500. A total of five highly divergent regions from SC regions were detected (**Figure 2A**), including three intergenic spacers (IGS) (*rps16-trnQ*, *petN-psbM*, and *rpl32-trnL*), and two coding genes (*rpl32* and *ycf1*). Among these hypervariable sites, the *ycf1* gene exhibited the highest Pi value (0.06292), and the lowest was from *rpl32-trnL* (0.05002). Overall, the IR regions exhibited less variation (**Figure 3B**), and divergence was greater in the non-coding region than in the coding region (**Supplementary Figure 3**).

### 3.3 Repeat sequence polymorphisms

Simple sequence repeats (SSRs) across the 28 Linderniaceae plastomes were detected, ranging from 49 (*Lindernia rotundifolia*) to 89 (*Vandellia montana*) (**Figure 3A**). Of these, mononucleotide, dinucleotide, and tetranucleotide SSRs were found in all taxa; trinucleotide repeats were not observed in *Bonnaya ruellioides*, *B. tenuifolia*, *Craterostigma nummulariifolia* and *Torenia benthamiana*; pentanucleotide repeats were not discovered in *B. antipoda*, *B. tenuifolia*, *Legazpia polygonoides*, *Lindernia procumbens*, *T. crustacea*, *T. oblonga*, *T. parviflora*, and *V. elata*; hexanucleotide repeats only were detected in *V. elata*, *V. scutellariiformis*, and *V. setulosa*. The most abundant type was mononucleotides, accounting for 74.82%, followed by dinucleotides with 11.87%, tetranucleotides with 9.32%, trinucleotides with 2.00%, pentanucleotides with 1.83%, and hexanucleotides with the least occurrence (0.17%) (**Supplementary Table 1**). The mononucleotide and dinucleotide SSRs primarily consisted of A or T bases and AT or TA bases, respectively, while the composition of other types of SSRs was diverse (**Supplementary Table 2**). Furthermore, most SSRs were distributed in the LSC region (**Figure 3C**), ranging from 71.43% to 87.72%, followed by the SSC region varying from 5.00% to 16.33%, and were rare in the IR regions, even were not detected in the IR region of *Picria felterrae* (**Supplementary Table 1**). The SSRs included between 55.56% and 78.33% in the IGS, between 11.11% and 30.16% in the exon, and between 8.16% and 21.05% in the intron (**Figure 3E** and **Supplementary Table 1**).

A total of 1909 long sequence repeats (LSRs) greater than 30 bp were identified, containing 844 forward repeats, 899 palindromic repeats, 107 reverse repeats, and 59 complement repeats (**Supplementary Table 3**). For each Linderniaceae species, the number of repeat sequences varied greatly, ranging from 42 (*Vandellia elata* and *V. megaphylla*) to 252 (*V. montana*) (**Figure 3B**). Among these long repeats, forward and palindromic repeats were detected in all species; reverse repeats were not recognized in *Craterostigma nummulariifolium*, *Lindernia procumbens*, *Torenia anagallis*, *V. megaphylla*, and *Yamazakia pusilla*; complement repeats were not discovered in seventeen individuals (**Figure 3D** and **Supplementary Table 3**). These LSRs were found predominantly in the LSC region (13.27–80.95%) and IR region (17.46–85.84%), with a few located in the SSC region (0.88%–5.45%) (**Figure 3D** and **Supplementary Table 3**). Meanwhile, most were detected in the IGS (18.58–82.54%) and exon (24.32–78.76%), with a few distributed in the intron (1.20–13.04%) (**Figure 3F** and **Supplementary Table 3**). Furthermore, the length of LSRs was mainly concentrated in 30–40 bp, accounting for 67.89%, followed by greater than 50 bp with 25.92%, and 41–50 bp with 16.19% (**Supplementary Table 3**).

### 3.4 Codon usage bias and selective pressure

Codon usage biases with high similarity were observed among 28 Linderniaceae species (**Supplementary Table 4**). The total number of codons of protein-coding genes ranged from 22,800 (*Picria felterrae*) to 22,895 (*Bonnaya antipoda*). Leucine (Leu) was the most abundant (2400–2434), while cysteine (Cys) showed the least abundance (240–252). The RSCU values of 30 codons were greater than 1, almost all of which ended with A or U except for UUG. Specifically, the UUA encoding Leu had the highest RSCU from 1.91 to 1.98, and the AGC encoding serine (Ser) had the lowest RSCU from 0.27 to 0.32. Methionine (Met) and tryptophan (Try) preferred one codon with RSCU = 1, namely AUG and UGG, respectively. To further evaluate the codon usage pattern, the GC content of synonymous third codon positions (GC3s) and the effective number of codons (ENC) were calculated in **Supplementary Table 5**. The values of ENC showed a slight bias ranging from 46.91 to 47.33. Total GC content in the codons was between 37.52% and 37.77%, and the values of GC3s varied from 28.7% to 29.2%. GC content was highest at the first codon position (45.67–45.94%), followed by the second position (38.18–38.39%), and third position (28.63–29.13%).

The ratios (Ka/Ks) of the non-synonymous (Ka) and synonymous (Ks) substitutions revealed that the plastid genomes of Linderniaceae were highly conserved during the evolutionary process (**Supplementary Table 6**). In terms of average values, the Ka/Ks values of all genes were less than 1 (between 0 and 0.7908), indicating that these genes were under purifying selection. Interestingly, the average Ka/Ks values of almost all genes were less than 0.5, except for *ycf2* (0.7908), *psbH* (0.6269), *ycf15* (0.5272), and *ycf1* (0.5241). Meanwhile, we found significant variation in the Ka/Ks values of genes with different functions. For example, photosynthesis-related genes were between 0 (*atpH*, *psaC*, *psaJ*, *psbE*, *psbF*, *psbL*, *psbM*, *psbN*, and *rps12*) and 0.6268 (*psbH*), self-replication genes ranged from 0.2300 (*clpP*) to 0.3139 (*matK*), other function genes varied from 0.02442 (*infA*) to 0.3927 (*accD*), and unknown function genes were from 0.0060 (*ycf3*) to 0.7908 (*ycf2*). Moreover, only the gene *ycf2* exhibited positive selection and was detected in six species, including *Bonnaya antipoda*, *B. ciliata*, *B. ruellioides*, *B. tenuifolia*, *Picria felterrae*, and *Torenia flava*. Four genes had Ka/Ks values equal to 0 in all species, namely *atpH*, *psaC*, *psbE*, and *psbF*.

### 3.5 Phylogenetic analyses

**Figure 4:**
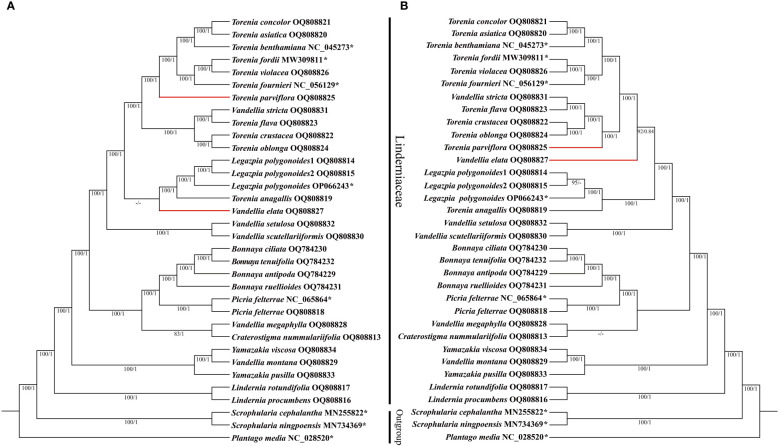
Cladograms of Linderniaceae inferred from the complete plastid genomes **(A)** and protein-coding genes **(B)** based on Bayesian inference **(BI)** and maximum likelihood (ML) methods. The ML bootstrap (BS) and BI posterior probability (PP) that supported each node are shown under the branches. Red-bold horizontal lines mean different phylogenetic positions for taxa between two different datasets. * indicates that the sequence is from GenBank. BS values < 50% and PP < 0.5 are indicated by ‘–’.

In this study, we included 31 complete plastomes from Linderniaceae and three outgroups from Plantaginaceae and Scrophulariaceae for phylogenetic inference. After removing ambiguously aligned regions, the alignment of the complete plastomes was 150,629 bp in length, comprising 11,541 parsimony-informative sites. A total of 80 protein-coding genes were aligned and the matrix of 79,174 bp was generated after removing ambiguously aligned regions, including 5,744 parsimony-informative sites. As topologies generated from ML and BI analysis were similar, only the ML tree was presented with ML bootstrap support (BS) and BI posterior probability (PP) values added (**Figure 4**). Except for the phylogenetic positions of *Vandellia elata* and *Torenia parviflora*, the topologies generated from the two datasets were identical. In the complete plastomes tree, *Vandellia elata* was sister to the group consisting of *Legazpia polygonoides* and *T. anagallis* with weak support (BS = 36, PP = 0.35). However, *Vandellia elata* was strongly supported as a sister to the core *Torenia* that comprised *V. stricta* and all *Torenia* species except *T. anagallis* in the protein-coding genes tree (BS = 92, PP = 0.84). *Torenia parviflora* was one of a member of the core *Torenia*. It was sister to the clade including *T. asiatica*, *T. concolor*, *T. fournieri*, *T. benthamiana*, *T. fordii*, and *T. violacea* in the complete plastomes tree (BS = 100, PP = 1.00) but sister to another group consisting of *V. stricta*, *T. flava*, *T. crustacea* and *T. oblonga* in the protein-coding genes tree (BS = 100, PP = 1.00).

In both trees, the monophyly of Linderniaceae was strongly supported (BS = 100, PP = 1.00). Within Linderniaceae, *Lindernia procumbens* was sister to *L. rotundifolia*, then together sister to the rest of Linderniaceae (BS = 100, PP = 1.00). The two *Yamazakia* species did not group together as *Vandellia montana* embedded within the genus (BS = 100, PP = 1.00). *Picria felterrae* from different populations clustered together and formed a sister relationship with monophyletic *Boyanna* comprising *Boyanna antipoda*, *B. ciliata*, *B. ruellioides*, and *B. tenuifolia* here (BS = 100, PP = 1.00). The monotypic *Legazpia* represented by three different individuals was strongly supported, which was sister to *Torenia anagallis* (BS = 100, PP = 1.00). The monophyly of *Torenia* was not supported with *V. stricta*, *V. elata*, and *Legazpia polygonoides* embedded. Similarly, the monophyly of *Vandellia* was also not supported as species of the genus scattered to other distinct clades.

## 4 Discussion

### 4.1 Conservatism and diversity of Linderniaceae plastomes

In this study, the 26 plastid genomes of Linderniaceae were newly sequenced, and comprehensive analyses were performed in combination with five published data. Comparative analyses revealed conservatism and diversity in the Linderniaceae plastomes. Similar to most angiosperms (Wicke et al., 2011), the plastid genomes of Linderniaceae typically presented circular quadripartite structure, comprising a pair of inverted repeats (IRs), a large single copy (LSC) region, and a small single copy (SSC) region (**Figure 1**). The 31 plastid genomes of Linderniaceae, ranging from 152,386 bp to 154,402 bp, did not show significant differences in size, and the gene content and order were identical (**Tables 2**, **3**). Within the same species, there is little or no variation in the genome size. The two populations of *Picria felterrae* differ in size by only 7 bases, and the three plastomes of *Legazpia polygonoides* had the same 153,477 bp in size (**Table 2**). Similarly, there are also no significant differences among distantly related species between *Torenia* and *Lindernia*, such as *Torenia concolor* (153,994 bp) and *Lindernia procumbens* (153,448 bp). These means that the plastid genomes of Linderniaceae are highly conservative in general features with only slight variations in genome size. Previous studies have revealed that the expansion and contraction of the IR, LSC, and SSC regions are common during evolution and are the main reason for variations in plastome size (Ravi et al., 2007; Downie and Jansen, 2015; Daniell et al., 2016). Significant contraction of IR region was observed among Linderniaceae species (**Supplementary Figure 2**), which was also common in Lamiales, such as Gesneriaceae (Gu et al., 2020), Lamiaceae (Su et al., 2022), Oleaceae (Long et al., 2023), Plantaginaceae (Xie et al., 2023), and Scrophulariaceae (Wang et al., 2022). Notably, the IR boundaries did not coincide exactly in the three populations of *L. polygonoides*, but they had the same plastome size. The two populations of *P. felterrae* were completely consistent in IR size, with slight variation occurring in the LSC and SSC regions. Overall, the 31 plastid genomes of Linderniaceae showed size differences mainly in the LSC region (**Table 2**). Consistent with previous studies (Yang et al., 2022; Bai et al., 2023; Peng et al., 2023), the non-coding and single copy (SC) regions were more divergent than the coding and IR regions (**Supplementary Figure 3**). We, therefore, speculated that the size variation of Linderniaceae plastomes was mainly attributed to the LSC region, especially in the non-coding regions, similar to the results of studies of Gao et al. (2022) and Li et al. (2023).

In Linderniaceae, the gene composition in each junction was almost identical, while the gene arrangement pattern was slightly different (**Supplementary Figure 2**). Interestingly, the gene arrangement pattern of each IR boundary in Linderniaceae was more similar among closely related species. For example, based on the phylogenetic analyses in this study, *Torenia crustacea*, *T. oblonga*, *T. flava*, and *Vandellia stricta* formed a well-supported clade (**Figure 4**), and their IR boundaries were highly conservative, with only slight differences in the SSC-IRb and SCC-IRa junctions. Particularly, the sister species of *T. crustacea* and *T. oblonga* showed completely consistent boundary genes and arrangement patterns, and so did the sister species of *T. flava* and *V. stricta*. This correlation has been reported in Aristolochiaceae (Bai et al., 2023), Juglandaceae (Xi et al., 2022), and Musaceae (Song et al., 2022). Therefore, information from the IR boundary may reveal the phylogenetic relationships among species to some extent.

Long sequence repeats (LSRs) play an important role in genome recombination and rearrangement (Do and Kim, 2017). In Linderniaceae, LSRs were found predominantly in the IR regions, and the forward and palindromic repeats with the length mainly concentrated in 30–40 bp were the most abundant (**Supplementary Table 3**). These findings were largely consistent with previous studies (Tian et al., 2018; Thode and Lohmann, 2019; Fan et al., 2021; Zhu et al., 2021). Single Sequence Repeats (SSRs) with polymorphism have been developed as molecular markers that were widely used in population genetics, polymorphism investigation, and biogeographic inference (Ebert and Peakall, 2009; Xue et al., 2012; Yu et al., 2017). In 28 Linderniaceae species, the mononucleotide SSRs were the most abundant and almost consisted of A or T bases (**Supplementary Table 2**), which may explain the richness of A/T content in plastomes. The number of SSRs in the single-copy (SC) and non-coding regions was higher than that in the IR and coding regions (**Figure 3**), consistent with previous research (Huang et al., 2020; Kong et al., 2021; Wu et al., 2021). There are significant differences in the number and distribution pattern of SSRs among species (**Figure 3** and **Supplementary Table 1**), which have great significance for future population genetics studies of Linderniaceae.

Hypervariable regions within the plastid genome have been screened out as candidate barcodes for phylogenetic analyses and species identification (Dong et al., 2012; Dong et al., 2015; Li et al., 2015). As non-coding sequences have higher evolutionary rates than coding regions, they were often identified as the best barcoding candidates (Ravi et al., 2007), which was confirmed in the present study (**Figure 2** and **Supplementary Figure 3**). Five hypervariable regions were screened within the Linderniaceae, namely *petN-psbM*, *rps16-trnQ*, *rpl32-trnL*, *rpl32*, and *ycf1*, of which *petN-psbM* and *rps16-trnQ* were located in the LSC region and others were distributed in the SSC region. These non-coding regions have exhibited high nucleotide diversity and phylogenetic utility in Lamiales, such as *petN-psbM* in Lamiaceae (Su et al., 2022; Wang et al., 2023), *rps16-trnQ* in Scrophulariaceae (Dong et al., 2022) and Gesneriaceae (Gu et al., 2020), and *rpl32-trnL* in Bignoniaceae (Fonseca and Lohmann, 2018), Lamiaceae (Hu et al., 2020) and Scrophulariaceae (Dong et al., 2022). Furthermore, the gene *ycf1*, the second largest gene in the plastid genome playing a critical role in plant cell survival (Drescher et al., 2000), has been proposed as the most potential DNA barcode due to its high variability in land plants (Drew and Sytsma, 2011; Dong et al., 2015). The relatively high nucleotide diversity observed in the *rpl32* gene is similar to that observed in Dioscoreaceae (Wonok et al., 2023), Fagaceae (Worth et al., 2019), and Poaceae (Guo et al., 2022). In the previous study, the phylogeny of Linderniaceae inferred from a few common plastid markers (*rps16*, *matK*, *trnL*-*F*, and *trnK*) or nuclear gene (*RPB2*) had moderate or weak support values (Fischer et al., 2013; Liang, 2017; Biffin et al., 2018), hindering the investigation for the phylogeny and evolution of Linderniaceae. Hypervariable regions in this study provided additional information for further phylogenetic and biodiversity research in Linderniaceae.

### 4.2 Evolution of protein-coding genes in Linderniaceae

Codon usage bias may help to better understand gene expression and genome evolution (López et al., 2019). The analyses of relative synonymous codon usage (RSCU) showed that the Linderniaceae plastomes were highly similar in codon usage pattern (**Supplementary Table 4**). Consistent with previous studies (Azarin et al., 2021; Zhao et al., 2022; Li et al., 2023), Leucine (Leu) was the most frequent amino acid, and cysteine (Cys) was the least common. In Linderniaceae, codons mainly ended with A and U when the RSCU value was greater than 1, and codons primarily ended with G and C when the RSCU value was below 1, which appears to be a common phenomenon in gene expression of land plants (Cui et al., 2019; Chakraborty et al., 2020; Gao et al., 2022; Bai et al., 2023; Zhou et al., 2023). Moreover, the GC content of synonymous third codons positions (GC3s) showed that AT content was more abundant in the protein-coding genes (**Supplementary Table 5**), which may be correlated with the abundant AT content of plastid genomes. The ENC values of protein-coding genes in Linderniaceae plastomes varied from 46.91 to 47.33, demonstrating a conservative codon usage pattern (Wu et al., 2007).

The ratio (Ka/Ks) of non-synonymous (Ka) and synonymous (Ks) substitutions is a valuable parameter to assess the selective pressure in evolution (Ohta, 1995). Positive selection is considered to be closely associated with adaptive evolution in harsh environments while purifying selection is a common evolutionary force responsible for maintaining genomic conservation (Cvijović et al., 2018). In Linderniaceae, the Ka/Ks values of almost all genes were less than 1.00, providing evidence for purifying selection. The *ycf2* gene had a positive selection in only *Bonnaya antipoda*, *B. ciliata*, *B. ruellioides*, *B. tenuifolia*, *Picria felterrae*, and *Torenia flava*, with a Ka/Ks value greater than 1, indicating that most protein-coding genes were relatively conserved. As the largest known plastid gene in angiosperms, *ycf2* is an enigmatic gene due to its unknown function. Drescher et al. (2000) and Kikuchi et al. (2018) pointed out that the *ycf2* gene is associated with plant viability and the 2-MD AAA-ATPase complex. Among many angiosperms, *ycf2* has been reported under positive selection (Liu et al., 2018; Zhong et al., 2019; Peng et al., 2020; Wu et al., 2020; Song et al., 2022; Fu et al., 2023). Overall, protein-coding genes of plastid genomes of the Linderniaceae were highly conserved during long-term evolution, while the functions of the gene *ycf2* in adaptive evolution need to be further verified.

### 4.3 Insights into the phylogeny of Linderniaceae

In this study, the robust phylogenetic backbone of Linderniaceae was constructed with the whole plastomes, in which the support values of these clades were greatly improved compared with previous studies based on a few genetic markers (Fischer et al., 2013; Liang, 2017; Biffin et al., 2018). Based on phylogenomic analyses presented here, we found that *Vandellia* and *Torenia* sensu Fischer et al. (2013) were polyphyletic. Meanwhile, strong evidence was provided to support the taxonomic status of two genera, *Picria* and *Yamazakia.*

Traditionally, the genus *Torenia* is characterized by winged calyx on the shallowly lobed calyx tube and completely calyx-enclosed capsule (Bentham, 1846). However, historically, it is very difficult to define the exact circumscription of *Torenia*. Some species formerly belonging to *Craterostigma*, *Lindernia* s.l., and *Vandellia* were once treated as members of *Torenia* (Bentham, 1846; Hance, 1868; Pennell, 1943). On the basis of synapomorphy of the calyx tube together with molecular evidence, Fischer et al. (2013) redefined the genus *Torenia* with a total of 51 species included. In the phylogenetic analyses, *Torenia* seemed to be monophyletic as the only two species sampled formed a sister relationship (Fischer et al., 2013). Subsequent phylogenetic analyses based on expanded sampling indicated that *Torenia* sensu Fischer et al. (2013) is not monophyletic as some species of *Vandellia*, *Legazpia*, and *Schizotorenia* are embedded within it (Liang, 2017). Combining morphological and molecular evidence, Liang (2017) lumped *Legazpia*, *Schizotorenia*, and some species with claviform appendages of *Vandellia* into *Torenia*, with the number of species expanding to 78. According to the taxonomic treatment of Liang (2017), the morphology of the calyx tube was no longer used as a generic diagnostic characteristic, and potential synapomorphies of newly circumscribed *Torenia* included the pinnate vein, claviform appendages at the abaxial stamens, and bothrospermous seed. In this study, phylogenomic analyses including 11 species of *Torenia* indicated that *Torenia* sensu Fischer et al. (2013) is not monophyletic with *Vandellia stricta*, *V. elata*, and *Legazpia polygonoides* embedded. Morphologically, *Legazpia* can be easily distinguished from other *Torenia* species by such characteristics as three large semi-circular wings on the calyx, glabrous ovary, small corolla slightly exceeding the calyx, and rounded upper corolla lip (Yamazaki, 1955), and has been accepted as a separate genus by most taxonomists (Yamazaki, 1955; Fischer, 1995; Hong et al., 1998; Fischer, 2004; Rahmanzadeh et al., 2005; Fischer et al., 2013; Fischer et al., 2013). To maintain the monophyly of *Torenia*, reducing *Legazpia* to *Torenia* may be a reasonable choice.

*Vandellia* has long been treated as a synonym of *Lindernia* s.l. (Pennell, 1935; Pennell, 1943; Philcox, 1968; Yamazaki, 1977; Yamazaki, 1985; Fischer, 1995; Lewis, 2000; Fischer, 2004; Rahmanzadeh et al., 2005). Based on molecular phylogenetic analyses, Fischer et al. (2013) revealed that *Lindernia* s.l. is polyphyletic and split this genus into six genera (*Lindernia* s.s., *Vandellia*, *Torenia*, *Bonnaya*, *Linderniella*, and *Craterostigma*), of which most species of *Lindernia* s.l. were assigned to *Vandellia*. In this study, phylogenetic analyses showed that the species of *Vandellia* sensu Fischer et al. (2013) scattered various clades, which was similar to previous studies (Liang, 2017; Biffin et al., 2018). Traditionally, the pinnate vein, deeply lobed calyx, and four fertile stamens were usually used to define the genus *Vandellia* (Bentham, 1846; Hooker, 1884; Fischer et al., 2013). On the basis of morphological together with molecular evidence, Liang (2017) transferred *Chamaegigas*, *Craterostigma*, and *Linderniella* which have been long-established and accepted genera to *Vandellia*, although the *Vandellia* clade had weak support based on combined DNA markers (*trnL-F*, *rps16*, and *matK*). *Vandellia* sensu Liang (2017) comprised 49 species, almost all of which were transferred from other genera. A large number of species name changes will lead to confusion for end-users of these names (most of them are not taxonomists) (Drew et al., 2017). Due to limited sampling in the current study, we have insufficient evidence to elucidate the circumscription of *Vandellia*, pending comprehensive phylogenomic analyses to better determine the strength of support for this genus.

The *Yamazakia* was currently proposed by Biffin et al. (2018) as a replacement name for the genus *Tittmannia* Rchb. (blocking name: *Tittmannia* Brongn.), including two sampled species in phylogenetic analyses (*Yamazakia pusilla* and *Y. viscosa*). These two species were transferred from *Vandellia* sensu Fischer et al. (2013). Actually, Liang (2017) has revealed a well-supported clade (BS = 100, PP = 1.00) including three species [*Vandellia moillis* (synonym of *V. montana*)*, V. pusilla*, and *V. viscosa*], and placed them in the genus *Tittmannia* together with other five species (*Tittmannia cyrtotricha*, *T. longituba, T. rivularis*, *T. satakei*, and *T. stolonifera*) based on morphological evidence. Traditionally, these species shared similar morphological characteristics and therefore were placed within the *Lindernia* section *Tittmannia* (Philcox, 1968). In our study, phylogenetic analyses showed that *V. montana* was sister to *Yamazakia viscosa* with strong support (BS = 100, PP = 1.00), then together sister to *Y. pusilla* (BS = 100, PP = 1.00). Resolution of the three species is significantly improved compared with the study of Liang (2017). Therefore, our results demonstrated *V. montana* to be a member of *Yamazakia*, supporting *Yamazakia* as a separate genus.

The genus *Picria* was proposed by Loureiro (1790), containing only one species *Picria felterrae*. The native range of this genus is Himalaya to S. China and Peninsula Malaysia, Philippines to Caroline Islands. *Picria* is a very distinctive genus morphologically different from the other Linderniaceae plants (Fischer, 2004). The calyx tube of Linderniaceae is composed of four types, namely deeply lobed, shallowly lobed, half lobed, and no calyx tube (Liang, 2017). The last type is in fact that the calyx is completely split to base and divided into four spreading segments, consisting of a pair of cordate outer sepals and another pair of filiform lateral inner sepals. This type of calyx tube only occurs in *Picria* within the Linderniaceae. Liang (2017) first included *Picria* in molecular phylogenetic analyses and indicated that the genus was sister to *Boyanna* with moderate support (BS = 67, PP = 0.64). In this study, we included two individuals from different populations in phylogenetic analyses. The result further confirmed that *Picria* is closely related to *Boyanna* with strongly supported values (BS = 100, PP = 1.00). Except for significantly different calyx morphology, the two genera bear similar corolla tubes, clavate staminodes without appendages, and flora discs, which may reflect the relationship between the two genera. Due to the particular calyx and relatively isolated phylogenetic position, we supported the monotypic *Picria* as a separate genus.

## 5 Conclusion

Linderniaceae plastomes were highly conservative in terms of structure, gene order, and gene content, with slight contraction in the IR region. Except for that the *ycf2* gene has a positive selection in a few species, most protein-coding genes were highly conserved, indicating that the evolution of Linderniaceae species was relatively slow. The phylogenetic analyses of Linderniaceae based on the complete plastid genome showed a higher resolution compared with previous studies, and the results revealed that the *Vandellia* and *Torenia* sensu Fischer et al. (2013) were non-monophyletic. The newly established *Yamazakia* was supported and the monotypic *Picria* could be regarded as a separate genus. Molecular phylogenetic evidence is of great importance to taxonomic revision of the complicated Linderniaceae. In this study, the sampling is not comprehensive with only 28 representative species from eight genera. As the plastid genome has been demonstrated to be effective in solving the phylogeny of Linderniaceae, a widely accepted taxonomic treatment is likely to be reached based on comprehensive sampling and plastid genome data.

## Data availability statement

The data presented in the study are deposited in the NCBI repository (https://www.ncbi.nlm.nih.gov/), and the accession numbers can be found in the article.

## Author contributions

GH conceived and designed the study. GH, RY, and XZ collected the samples. RY and YJ performed experiments and data analyses. RY and GH drafted the manuscript. GH, CX, YG, and RY revised the manuscript. All authors contributed to the article and approved the submitted version.
