# Supplementary figures and images for: Comparative analyses of Linderniaceae plastomes, with implications for its phylogeny and evolution
Source: Front Plant Sci. 2023 Sep 26;14:1265641. doi: 10.3389/fpls.2023.1265641 (PMC10565954; doi:10.3389/fpls.2023.1265641)

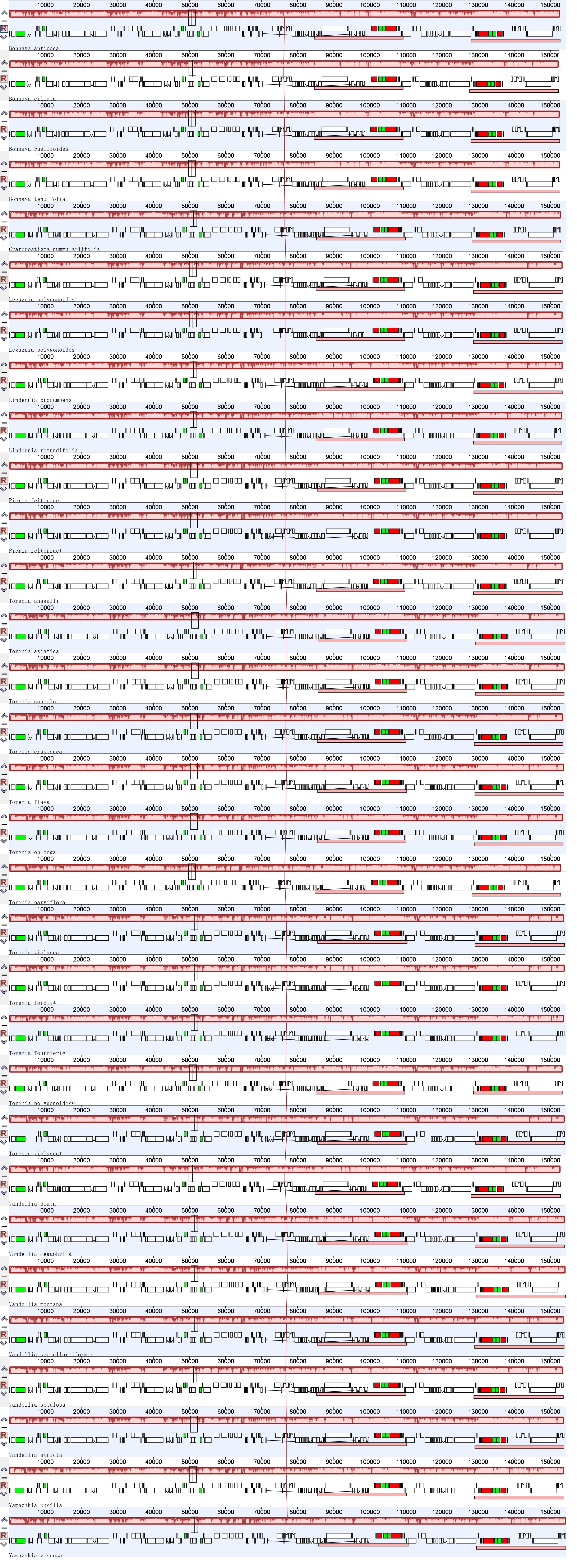

Supplement: Supplementary file 1 [file DataSheet_1.zip › Supplementary material/Supplementary Figure 1.jpg]

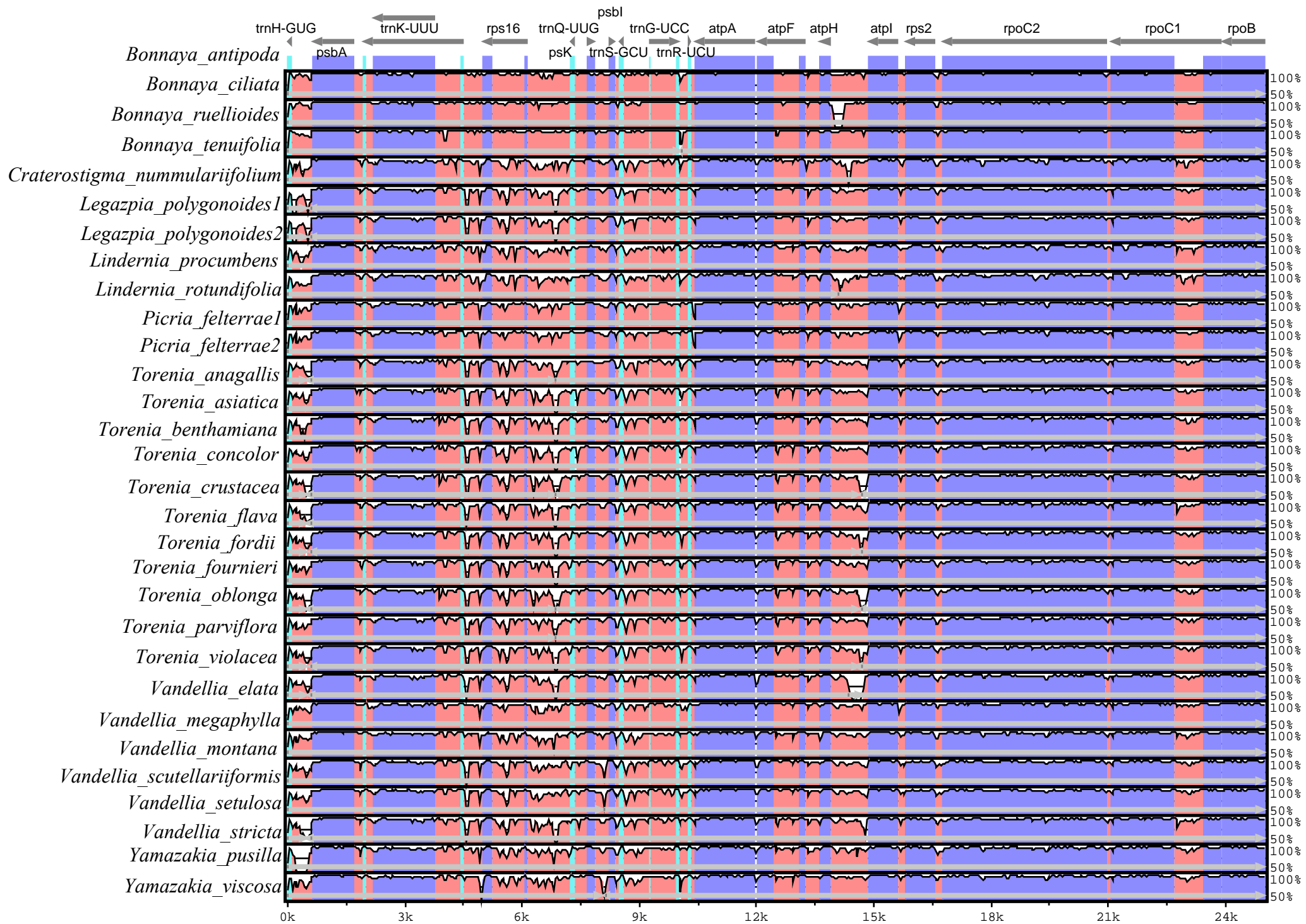

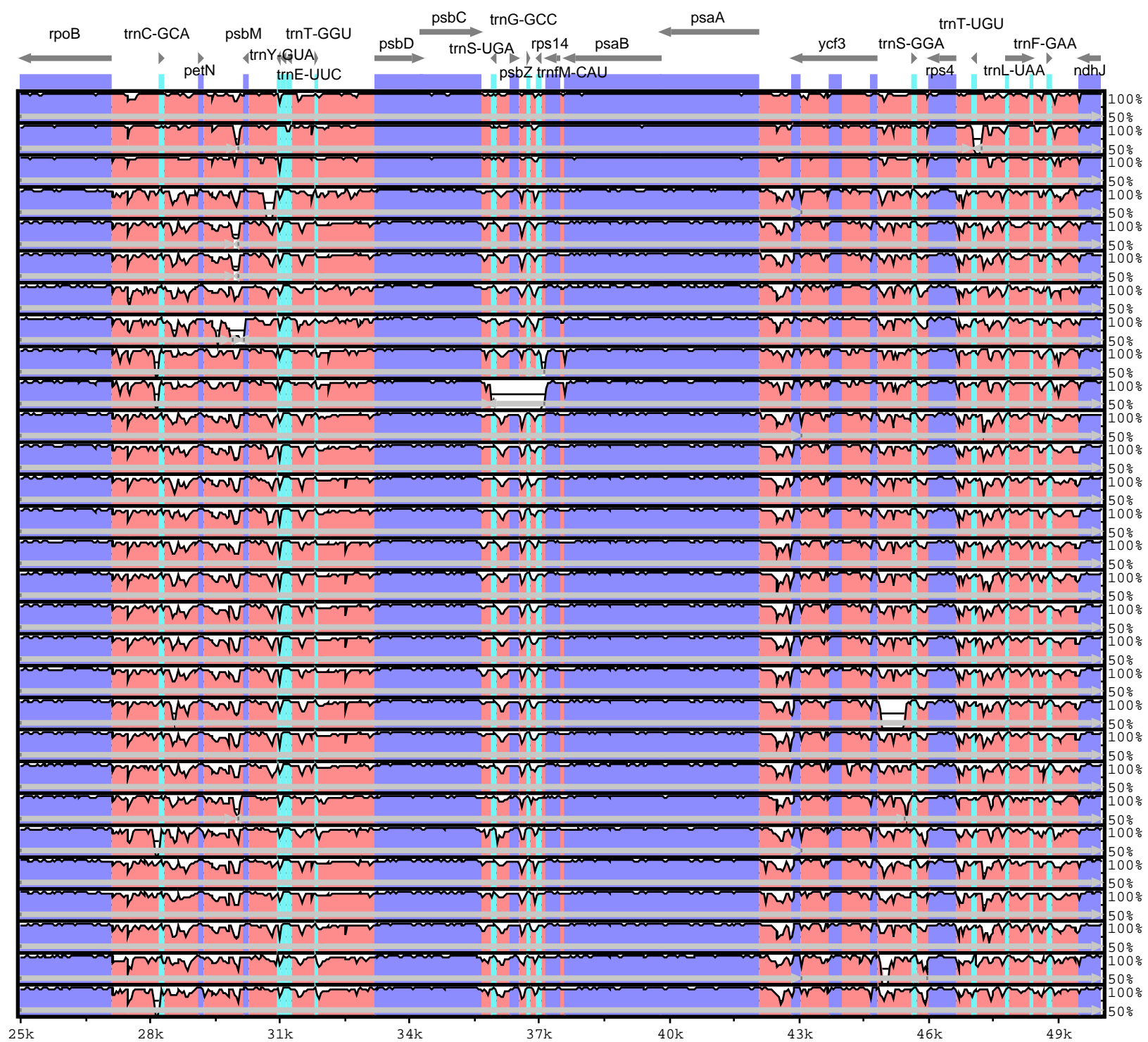

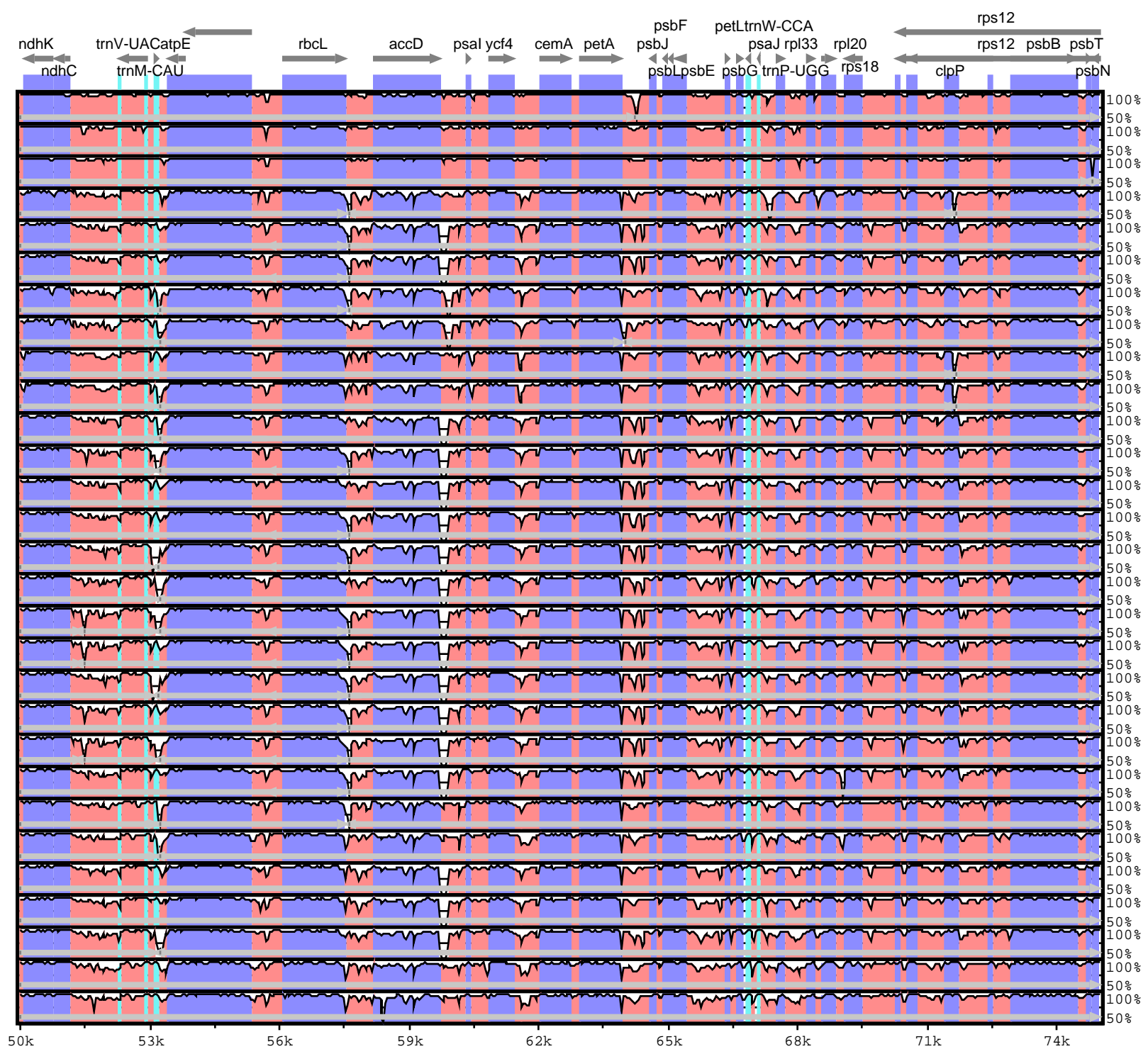

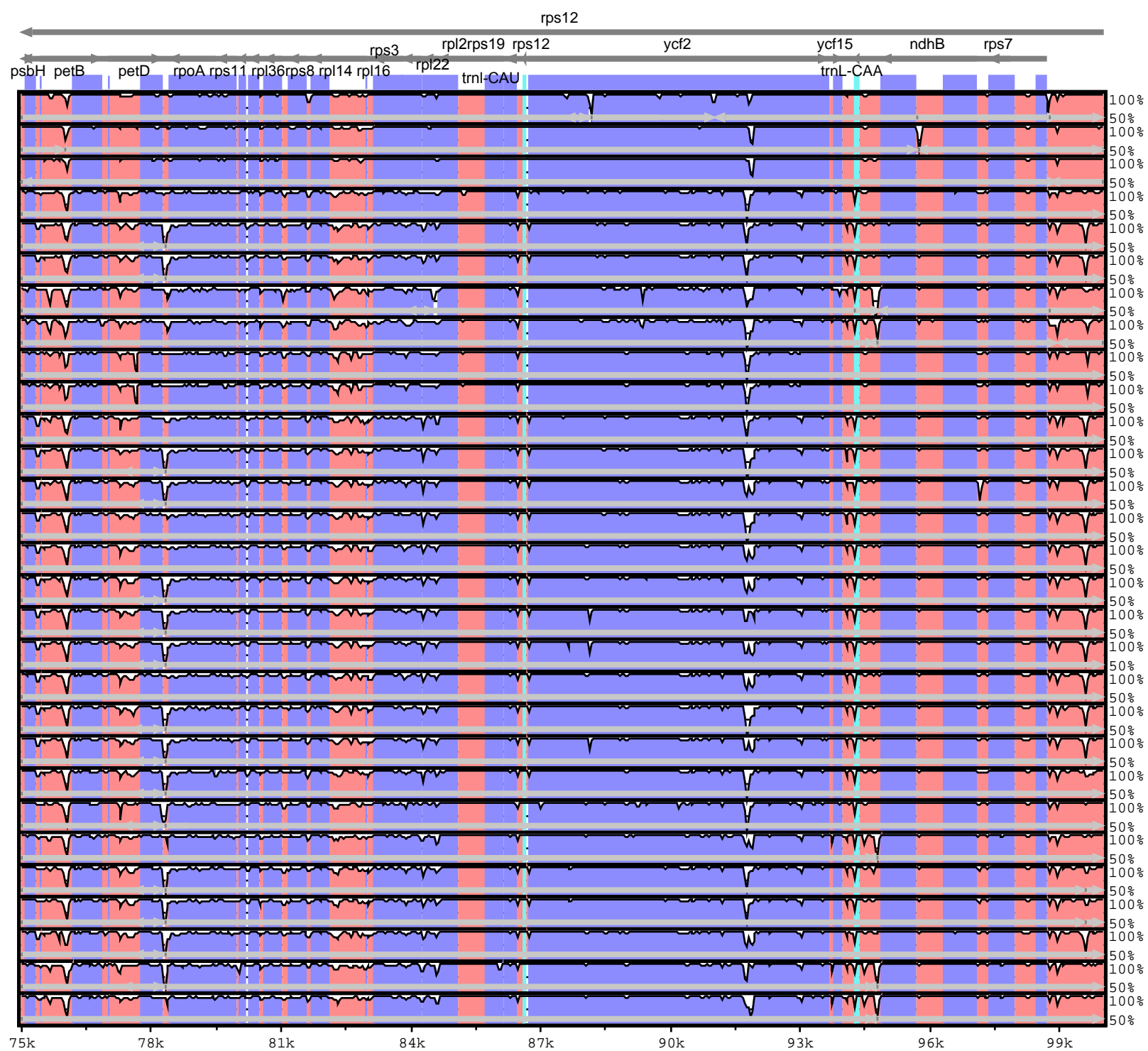

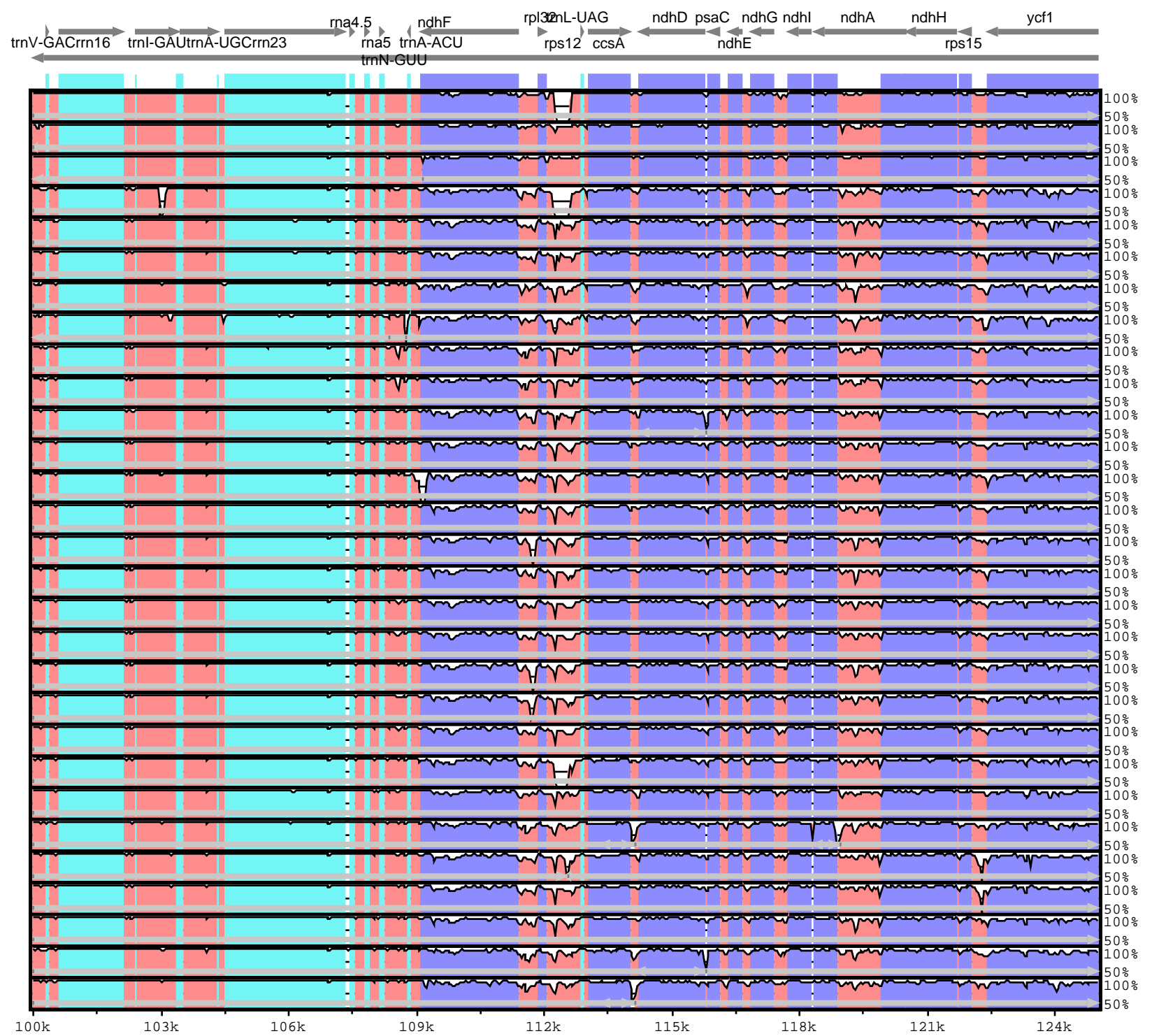

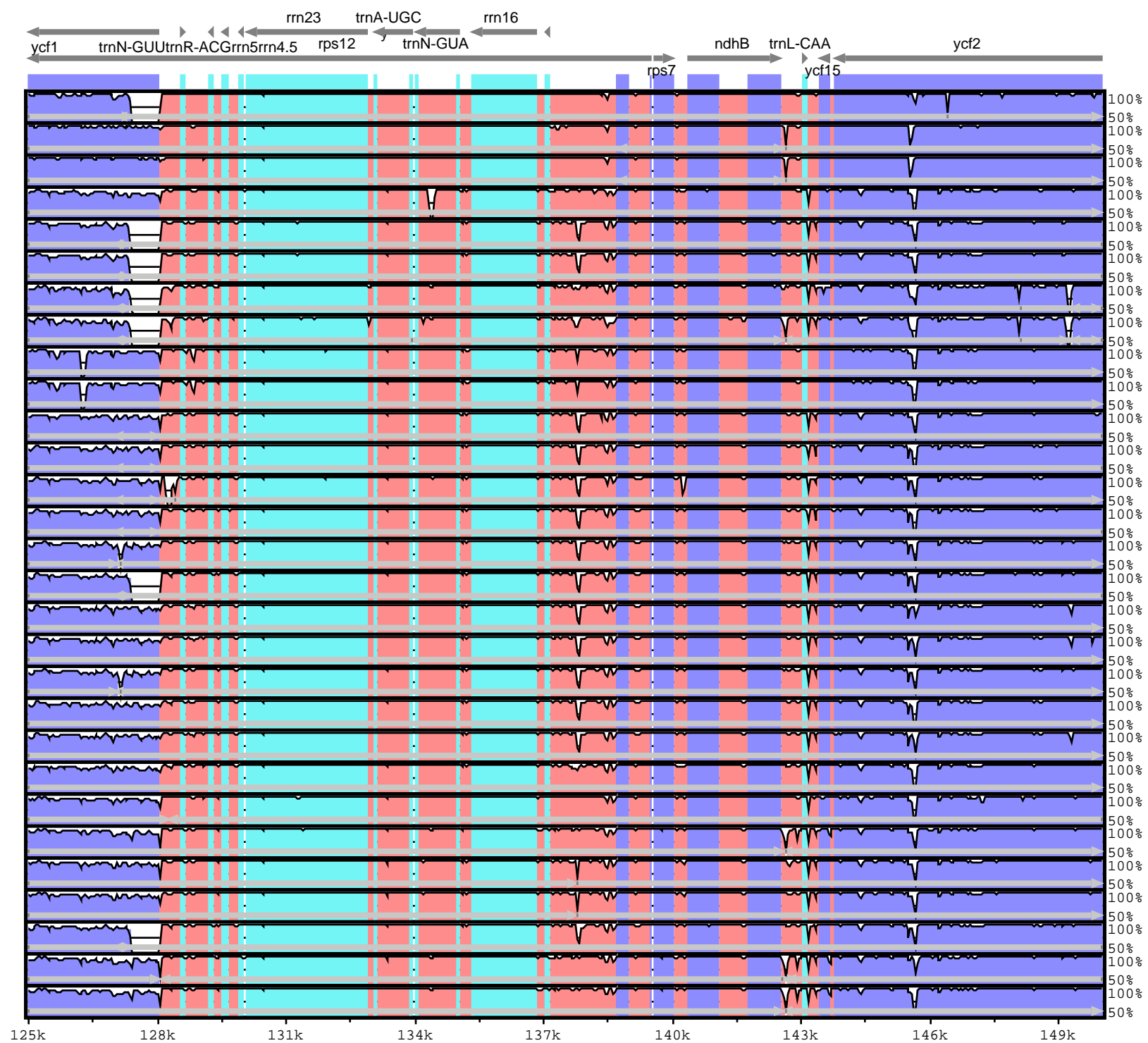

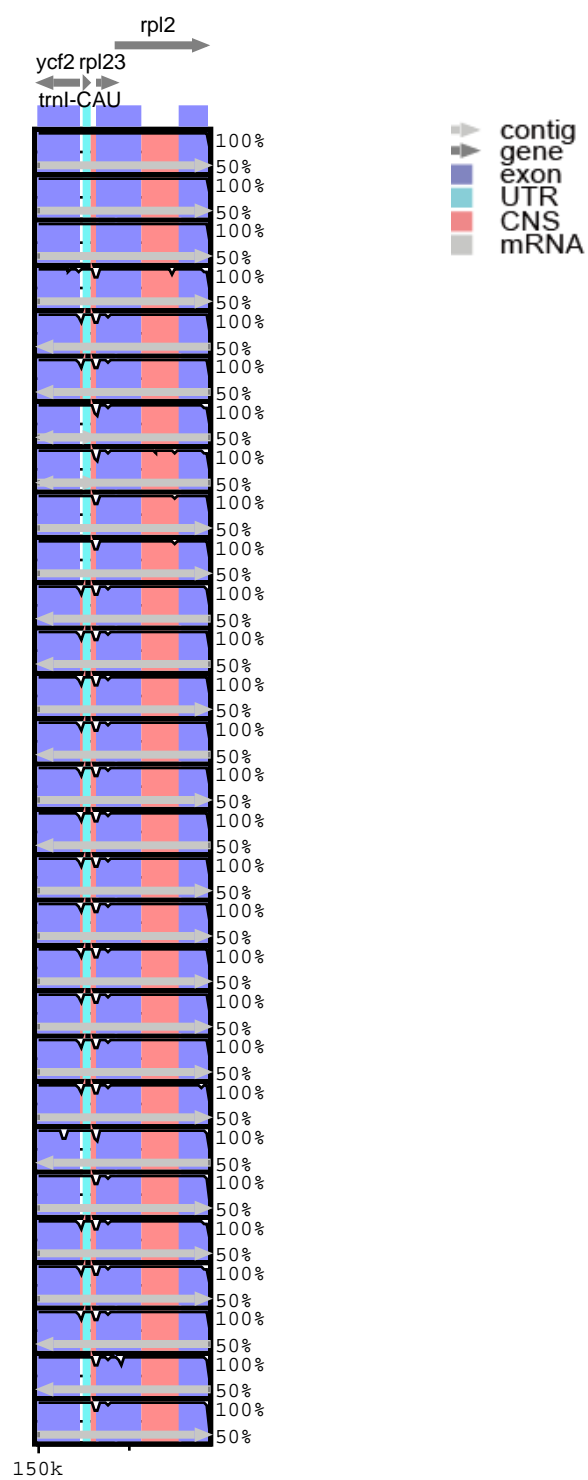

Supplement: Supplementary file 1 [file DataSheet_1.zip › Supplementary material/Supplementary Figure 3.pdf]
